# Shoe-Insole Technology for Injury Prevention in Walking

**DOI:** 10.3390/s18051468

**Published:** 2018-05-08

**Authors:** Hanatsu Nagano, Rezaul K. Begg

**Affiliations:** Institute for Health and Sport (IHES), Victoria University, Melbourne, VIC 3032, Australia; hanatsu.nagano@vu.edu.au

**Keywords:** gait, insole, injury prevention

## Abstract

Impaired walking increases injury risk during locomotion, including falls-related acute injuries and overuse damage to lower limb joints. Gait impairments seriously restrict voluntary, habitual engagement in injury prevention activities, such as recreational walking and exercise. There is, therefore, an urgent need for technology-based interventions for gait disorders that are cost effective, willingly taken-up, and provide immediate positive effects on walking. Gait control using shoe-insoles has potential as an effective population-based intervention, and new sensor technologies will enhance the effectiveness of these devices. Shoe-insole modifications include: (i) ankle joint support for falls prevention; (ii) shock absorption by utilising lower-resilience materials at the heel; (iii) improving reaction speed by stimulating cutaneous receptors; and (iv) preserving dynamic balance via foot centre of pressure control. Using sensor technology, such as in-shoe pressure measurement and motion capture systems, gait can be precisely monitored, allowing us to visualise how shoe-insoles change walking patterns. In addition, in-shoe systems, such as pressure monitoring and inertial sensors, can be incorporated into the insole to monitor gait in real-time. Inertial sensors coupled with in-shoe foot pressure sensors and global positioning systems (GPS) could be used to monitor spatiotemporal parameters in real-time. Real-time, online data management will enable ‘big-data’ applications to everyday gait control characteristics.

## 1. Introduction

Walking is a fundamental locomotor task essential to healthy, active living, but it is accompanied by injury risk, particularly among the senior population. Walking is a continuum of gait cycles repeated thousands of times daily, and suboptimal features of the gait cycle can increase the probability of injury. Older adults (particularly females), for example, are prone to falls-related acute injuries due to gait impairments [1,2], and impaired foot pressure control during the loading response can cause foot problems [3]. The knee joint can be also affected over time due to functionally undesirable weight bearing, possibly resulting in osteoarthritis (OA) [4]. While walking is critically important both from a functional perspective and to ensure adequate exercise, injury risks must be minimised by optimising some essential biomechanical features of the gait pattern. Biomechanical interventions for injury prevention should, however, fulfil certain practical requirements, including low cost, easy engagement, immediate effects, and little physical effort; otherwise, interventions are unlikely to be adopted voluntarily and maintained in the longer term [5]. Footwear interventions have the potential to satisfy these requirements.

Typically, shoes are constructed with a number of components, all of which can influence gait mechanics [6]. An elevated heel is a factor in lateral instability and may result in caution-related adaptations reflected in spatio-temporal parameters [6]. Compared to standard soles, hard soles can more effectively provide tactile sensation for quicker reactions to maintain balance. Footwear-collars improve balance due to increased tactile sensation around the ankle while reducing swing foot clearance [6]. The outsole provides the interface with the walking surface and affects the frictional demands of walking and associated risk of slipping [7]. In contrast, the insole has direct contact with the sole of the foot and directly controls foot pressure and ankle joint motion that, in turn, influences the individual’s gait pattern [8]. While some features of ‘safe shoes’ tend to be avoided, such as firm shoe-lace fixation, particularly in individuals with impaired activities of daily living (ADL) [9], insole interventions have the potential to be more readily accepted due to their practicality when applied to various footwear types.

As summarised in Table 1, three main types of insole modification can be identified as having the potential to support safer walking. Until recently, most high-grade insoles were produced using custom-moulding, which was designed to accommodate the individual’s foot shape and influence foot pressure distribution. While this approach has provided a springboard, sensor technologies are now available that can provide a highly detailed biomechanical analysis of foot pressure and gait patterns to considerably advance shoe-insole development. Such progress could revolutionise injury prevention. For example, three-dimensional (3D) motion capture systems (e.g., Optotrack, Vicon, Optitrack) can accurately model gait motions, which is useful for identifying suboptimal gait features. By utilising this sensor technology, insole development can be undertaken to optimise gait control.

Foot pressure mapping, another very useful in-shoe sensor technology (e.g., F-scan, Pedar), is often synchronised with 3D motion capture to reveal the foot’s pressure distribution and centre of pressure (CoP) in real-time [10]. The advantages of insole sensor systems are portability and wireless communication. Foot pressure measurement can not only be utilised in developing new insoles but also has the potential to acquire and store data to record gait patterns. Body-mounted inertial sensors are a related technology with similar potential to sample gait parameters in the natural environment. The portability of sensor-based systems will be their essential advantage in future gait assessments and will gradually overcome the limitations of laboratory-based 3D motion capture systems.

In the current review, typical locomotor injuries are first explained and then insole developments and their significance are thoroughly discussed. Finally, the concept of a wireless gait measurement insole will be introduced and future directions in gait-related sensor technology outlined.

## 2. Biomechanics of Locomotive Injuries Based on Gait Analysis

In daily locomotion, both acute and overuse injuries should be considered. The primary cause of acute injury during locomotion is falls, particularly in the older population [1]. In contrast, certain types of ankle and knee pathology can be classified as overuse injuries due to the accumulation of negative gait features over time rather than a single traumatic event. This section introduces the biomechanics of falls and lower limb injuries primarily at the ankle and knee joints.

### 2.1. Falls

Approximately 33% of senior adults fall annually and up to 20% of cases lead to serious injuries [1,2,19]. Falls in this context can be defined as “an unintentional coming to the ground or the lower level due to balance loss” [20]. Biomechanically, falls occur when balance is disturbed and not able to be restored, with the consequence that the individual makes forceful contact with either the walking surface or surrounding objects. This twofold process comprises, therefore, (1) an event that disturbs balance and (2) failure in balance recovery.

**Figure 1:**
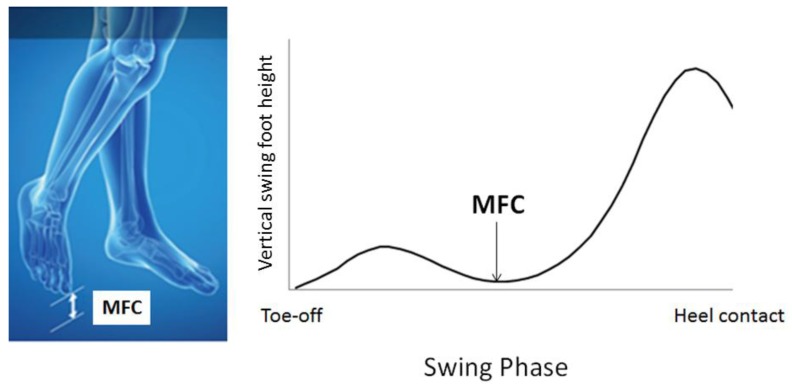
Minimum Foot Clearance (MFC).

Of the many balance-disturbing events, tripping has been identified as the leading cause of falls, accounting for up to 53% of all falls [21]. Tripping is due to physical contact of the swing foot with the walking surface or an object on it, which creates the momentum to significantly destabilise balance. To prevent tripping, therefore, swing foot clearance should provide a sufficient vertical margin, particularly at the mid-swing event—Minimum Foot Clearance (MFC)—illustrated in Figure 1. At MFC, the vertical margin of the swing foot from the walking surface is low (i.e., 1–2 cm) while moving at maximum speed and, as a consequence, causes high impact in the case of tripping [22]. Previous research by Moosabhoy and Gard suggests that, to prevent tripping, ankle dorsiflexion should be the most effective lower limb joint control strategy, whereby one degree of ankle dorsiflexion at MFC can be predicted to elevate the toe by .3 cm [23], a response that can significantly reduce the probability of tripping [24].

Biomechanically, dynamic balance is defined by the relationship between body centre of mass (CoM) and base of support (BoS) [25]. In gait analysis, sensors, such as infrared light-emitting diodes (IREDs), light-emitting diodes (LEDs), and passive reflective markers, are usually attached to anatomical landmarks to model the subject’s motion relative to a pre-registered laboratory coordinate system (*x*, *y*, *z*). In static conditions, when the CoM is preserved within BoS, balance is considered to be secure, whereas in dynamic conditions, including walking, extrapolated CoM (XCoM) is used rather than CoM, as in the equation below [26].

XCoM=CoM position+CoM velocitygravityl

**Figure 2:**
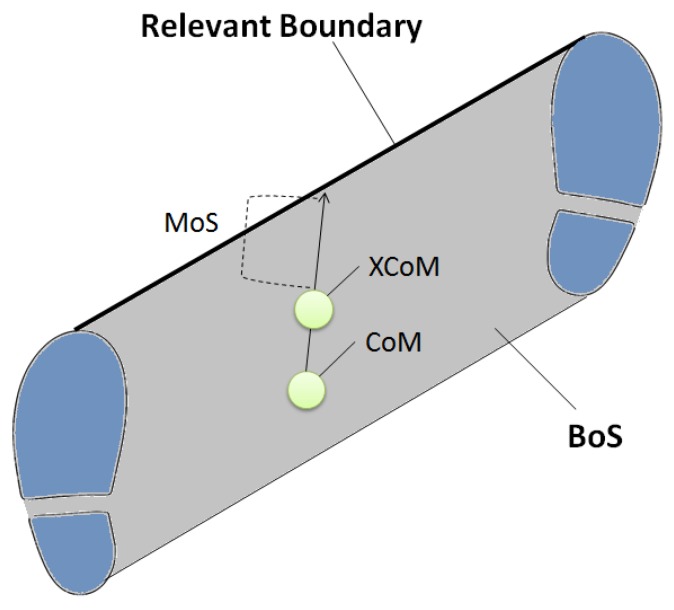
Description of balance in the transverse plane. MoS = margin of stability; BoS = base of support; CoM = centre of mass; XCoM = extrapolated centre of pass; Relevant boundary depending on the direction of CoM movement.

In the above equation, *l* indicates the distance between the ankle and the end of inverted-pendulum movement, CoM [27]. XCoM has been considered to more accurately represent the positional threshold within BoS because CoM position by itself does not differentiate static CoM from fast moving CoM. The distance between XCoM and either the lateral or anterior-posterior boundary of the BoS, depending on CoM movement direction, is defined as the margin of stability (MoS) (Figure 2). A greater MoS indicates that balance is secure while a negative (<0) MoS indicates balance loss and that the body is falling. From an injury prevention perspective, the most important feature of these relationships is that CoM motion is highly dependent on foot CoP control [28], which, as described above, can be influenced by footwear manipulations.

### 2.2. Foot-Related Conditions

Older adults’ footwear often has inappropriate features, perhaps due to prioritising comfort over safety [9]. This is reflected in the tendency to avoid shoes with fixation or heel counter, and sandals are commonly worn (22%) when they experience falls [9]. Improper footwear structures that cause undesirable foot pressure distribution and ineffective impact distribution can lead to chronic pain over time, which discourages active walking [29]. Footwear, including the insole, should be designed to promote walking mechanics that do not impose excessive stress on the foot and lower limb joints [3]. Foot problems often arise from inadequate weight bearing, which is reflected in undesirable ankle motion and foot pressure distribution [30,31]. Pressure distribution is the important element of gait control in preventing conditions, such as ulcer development, around the plantar areas, particularly among the diabetic population [32]. Fifteen million people in the U.S. have diabetes and 15–20% of those experience hospitalisation [3]. Another consequence of inadequate foot pressure distribution is Hallux valgus, which is especially common among high-heel wearers [14,33]. Hallux deformities are one of the major causes of foot pain [3]. Control of foot pressure is reflected in CoP movement, which is not only responsible for foot disorders but also controls dynamic balance. Inversion ankle sprain, for example, is a common acute injury caused by excessively lateral CoP [34], while Hallux valgus is a result of overpronation during mid to late stance [35]. As reported in previous studies [13,17] and as shown in Table 1, insole modifications can alter CoP control and foot pressure distribution, possibly reducing the incidence of debilitating foot conditions caused by inadequate CoP and foot pressure control.

Foot segment orientation is controlled by ankle motion, including dorsiflexion/plantarflexion and eversion/inversion. Pronation is the combination of dorsiflexion and eversion while inversion and plantarflexion form supination [36]. For adequate loading, the foot should be pronated in the early stance phase to absorb impact while quickly accommodating the walking surface environment [36,37]. Foot contact sometimes occurs with a supinated ankle but immediately starts to pronate, the essential countermovement to maximise the range of pronation, with accompanying tibial internal rotation [36,38]. Towards toe-off via foot-flat, the foot supinates, controlled first by eccentric work until foot-flat followed by the concentric work of supinators approaching toe-off [36,39]. This series of functional ankle motions most efficiently oscillates foot contact impact through the stance phase and into forward progression. These ankle motions are responsible for efficient weight transfer but also affect CoP movement, which can be measured using either force plates or in-shoe foot pressure monitoring systems.

**Figure 3:**
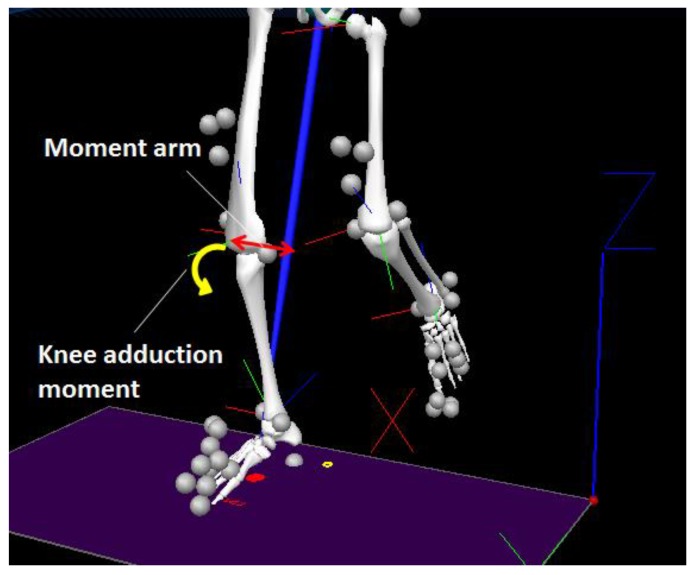
Illustration of marker setup for gait analysis; knee adduction moment due to shank external rotation.

Ankle kinematics can be monitored using 3D motion capture systems. For modelling the foot, a typical marker setup includes the toe (the distal and superior surface of the foot), second metatarsal head, fifth metatarsal head, lateral and medial malleolus, and heel. To visualise ankle motion, the tibia should also be modelled by incorporating lateral and medial epicondyles in addition to the lateral and medial malleolus at the ankle (Figure 3).

### 2.3. Knee Osteoarthritis

Knee osteoarthritis (OA) is a painful condition that discourages people from walking and increases the risk of falling [39]. OA is frequently associated with pain caused by micro-fractures due to bone-to-bone collision with reduced articular cartilage. Knee adduction moment is the kinetic marker most reliably related to the progression of OA, especially at the medial compartment [40,41]. A comprehensive review suggested that approximately 10–15% of the senior population show clinical evidence of OA [42], but it is possible that gait modification interventions using insoles slow the progression of OA if knee adduction moment can be reduced [43].

There are two peaks in knee adduction moment during a gait cycle. The first peak is associated with OA and timing approximates the first Ground Reaction Force (GRF) peak and maximum knee flexion [44]. Reducing knee adduction moment is, therefore, the challenge in OA prevention. Biomechanically, this can be achieved by (1) reducing GRF and (2) creating a shorter moment arm by reducing external shank rotation in the frontal plane after foot contact [45,46]. Shock absorption at the heel could reduce peak GRF, while tibial realignment in early stance is associated with reduced moment arm and knee adduction moment. Biomechanical gait data obtained using a combination of 3D motion capture systems and force plates (i.e., kinematic and kinetic analysis) can compute knee joint kinetics via an analysis technique known as inverse dynamics. As introduced later, a lateral wedge insole may be able to control CoP and realign the tibia to reduce knee adduction moment.

In summary, safe walking is essential to maintaining mobility without injury and, as shown in Table 1, shoe insoles can control gait functions to optimise walking mechanics [8,47]. In the current review, the primary focus is insole modifications to improve gait function.

## 3. Shoe Insole Technology for Gait Control and Injury Prevention

Shoe-insoles provide the interface between the foot and the footwear. Potential modifications include (1) modifying insole geometry (i.e., changing ankle angle) [18]; (2) increasing contact area (i.e., custom-moulding) [11]; (3) adjusting resilience (i.e., lower for shock absorption and greater for reusing mechanical energy) [12]; (4) treating insole surface (e.g., texture installation) [48]; (5) providing assistive support (e.g., heel counter) [49]; and (6) incorporating portable sensor systems, such as pressure sensors or inertial sensors [50]. Fundamental functions relating to reducing the above injury risks are as follows.

### 3.1. Shock Absorption

Impact energy at heel contact can be dissipated to minimise soft tissue damage, and softer materials are likely to reduce shock more effectively [51]. Ethylene vinyl acetate (EVA) foam is one of the more common shoe-insole materials, is usually used with a density range between 300 and 400 m/s^3^, and is ideal for semi-customised shoe-insole moulding in commercial production [52]. The choice of insole material characteristics, such as density, can be used to control elasticity and resilience.

Technology for shock absorption at the heel has been marketed commercially using materials such as gels. One biomechanical explanation for the effectiveness of shock absorption products is that they prolong the time from initial foot–ground contact until complete compression of the footwear. Although GRF and foot pressure distribution can be modified by construction materials [53], some studies have reported that shock absorbers may not reduce injury risks [54]. In addition to shock absorbing materials, insole structure and hardness of the mid-sole are important for reducing load [51,55].

Shock absorption utilising either a material’s elastic properties or a spring mechanism may be effective in storing mechanical energy at impact and then recovering the energy later in the stance phase toward toe-off as demonstrated by Zhang et al. using spring-loaded Axillary clutches [56]. Energy recovery can therefore engender more efficient walking by minimising the energy required at push-off. As detailed further in the following Section 3.2. Ankle, foot–ground impact can be viewed as an unexploited source of external energy because in human walking only 60–70% of impact energy can be oscillated through the loading response while the remainder is lost as vibration, sound, or heat [57,58].

### 3.2. Ankle

Ankle motions responsible for shock absorption during early loading are a combination of dorsiflexion and eversion (i.e., pronation) [36,59,60,61]. Flat-foot contact is not considered desirable for shock absorption because the interval from initial foot contact to foot flat determines impact distribution over time due to the eccentric work of dorsiflexors [37,38,61]. Ageing appears to be a factor in reduced dorsiflexion at foot contact [62].

A pronated ankle during early loading rotates the shank internally and triggers knee flexion [63,64,65], but both these kinematic adaptations have the potential to reduce knee adduction moment [51]. An over-pronation problem arises following mid-stance when the ankle should begin to supinate [36]. Given the anatomical constraints on foot kinematics during the stance phase, during early-loading foot orientation for shock absorption is important and after mid-loading over-pronation should be avoided.

Stored mechanical energy should be transferred throughout the loading response from the pronated ankle up to supinated orientation at mid-stance towards toe-off [36]. Quantification of mechanical energy transfer is possible by calculating the recovery rate (see below), the percentage of mechanical energy transferred to oscillate the loading response from heel contact to toe-off [64,65,66].

Recovery Rate (%)=100×[ΔKE+ΔPE−Δ(KE+PE)]/(ΔKE+ΔPE)

where ΔKE = increase in kinetic energy; ΔPE = increase in potential energy; Δ(KE + PE) = increase in the sum of kinetic energy and potential energy, all measured in the double support phase of the gait cycle based on CoM kinematics.

Step-to-step CoM transition during double support requires mechanical energy to produce GRF to continue walking [67]. More efficient loading is possible by utilising the impact at heel contact to initiate toe-off without dispersing the mechanical energy transferred to other lower limb joints (i.e., the knee). A higher recovery rate is, therefore, considered advantageous in reducing the heel contact forces transferred to other lower limb joints. For efficient loading, insole geometry can be re-designed to support pronation of the subtalar joint at heel contact while ensuring no disturbance to later ankle supination [36,59,60,61,68]. In the presence of foot deformity or other pathological conditions, however, careful consideration is required prior to any insole modification. For example, a custom-moulding insole modification may be necessary for seriously deformed feet, such as deformation due to neuropathy in diabetic patients [69]. Levinger et al. [45,46] suggested that a lateral wedge insole may be effective in reducing knee adduction moment by supporting pronation during early stance. They have, however, also stated that a lateral wedge insole may not be suitable for an already pronated foot.

### 3.3. Foot Pressure Control

The most common method for foot pressure distribution by shoe-insoles is to increase the contact area between the foot and the insole surface [70]. This can be achieved by custom-moulding the shoe insole to accommodate differences in foot shape [11]. Semi-customisation is also possible using an insole surface material that gradually adapts to the foot’s shape. Foot CoP trajectory in a normal gait starts at the heel and ends at the toe, with a lateral curvature [71]. This lateral excursion seems to be a reflection of ankle supination during mid-stance.

**Figure 4:**
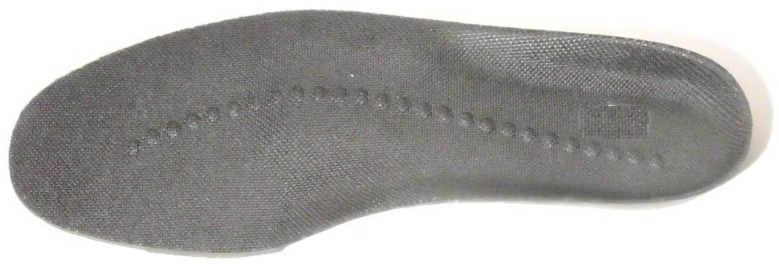
Example of texture installation to guide the CoP (WO2016015091A1—Injury Reduction Insole).

As indicated above, balance control is determined by the relationship between CoM and BoS. A specially designed insole can control the CoP which changes CoM motion and it is particularly important to regulate excessive lateral CoP excursion to stabilise sideways balance [59]. A potential solution is an insole incorporating enhanced texture to consistently guide the optimal CoP path (Figure 4).

If the CoP is variable over multiple gait cycles, associated CoM motions could also become variable and dynamic balance control could become unstable. Ensuring adequate cutaneous stimulation to assist the optimal CoP path is, therefore, a possible approach to improving balance. As described below, texture installation may also assist balance control by improving the reaction speed to balance perturbations.

### 3.4. Reaction Speed

Stimulation of cutaneous receptors increases afferent feedback and may, therefore, decrease reaction time. Ideally, the CoP should travel through (i.e., stimulate) high sensitivity cutaneous receptors without excessive pressure [72] and if balance is lost, recovery can be faster. Stimulation of the plantar surface is possible either by incorporating enhanced texture or using vibration devices [15,73]. The findings from these previous studies of this manipulation, however, leave some doubt as to how effectively such augmented stimulation can enhance afferent feedback and proprioception [15,73,74].

Priplata et al. [15] tested the effect of vibrating insoles on balance control and found that older adults had improved dynamic balance due to plantar stimulation, but the study methodology was questioned by Lafond et al. [74]. One of their primary concerns was the methods employed for estimating balance, which is a more general problem arising from the lack of standardised biomechanical analysis methods to evaluate footwear effects on gait and balance. Similarly, some studies have reported enhanced proprioceptive reaction effects of textured insoles [73,75,76], while others have not [77], and these discrepancies may, again, have been due to methodology but also to the fact that the textured or vibration insoles tested were not the same [78]. If cutaneous receptors are stimulated more systematically rather than stimulated by randomly installing textures over the entire insole surface, gait and balance could be controlled more effectively [78]. A further methodological limitation of previous insole research has been sample size; however, an ongoing study by Hatton et al. [79] with a large sample is expected to reveal the effects of texture stimulation on gait patterns with greater confidence.

There are a few reports of texture installation that is not applied over the entire surface. For example, SoleSensor is a commercially available shoe-insole that takes advantage of tactile stimulation to enhance reaction speed [16] using only a narrow tube peripheral to the insole surface. When foot pressure shifts peripherally and balance is disturbed, stimulation is provided by the tube that increases reaction speed. Similarly, Ritchile et al. applied textures only to the medial portion of the insole and reported that it supported functionally important mid-foot supination [68]. Due to the variety of texture installations with respect to area, size, shape, and hardness, further research is necessary to confirm the most effective stimulation properties. Research evidence suggests, however, that stimulation of cutaneous receptors has considerable promise for enhancing gait and balance [78].

### 3.5. Lateral Wedge Insole

The lateral wedge insole is designed to stabilize the ankle in a more everted position, helping to more internally align the tibia [46]. This adaptation reduces the tibial moment arm from the GRF vector and consequently decreases knee adduction moment [40,41]. For the Varus deformity, an everted ankle reduces knee adduction moment, softening the compression of the medial knee structure between the femur and tibia. The degrees of eversion support are usually between 5° and 15° [80].

Despite these biomechanical theories, the lateral wedge insole effects on knee OA are controversial. Weinhandl et al. [41], for example, reported no apparent effects in a young group who wore a lateral wedge insole for one week. As with the stimulation insoles, the conflicting results may be attributable to differences between the lateral wedge insoles in each study. Paradoxically, if the optimum structure can be identified, lateral wedges appear to have potential as a knee OA treatment [81]. Sawada et al. [82] reported that an individual’s foot alignment also determines the effectiveness of a lateral wedge insole in reducing peak knee adduction moment. Walking speed was also suggested to affect experimental results in terms of knee adduction moment, but this hypothesis has been rejected by one study [83]. An additional modification that may enhance the lateral wedge insole is arch-support [84]. Based on data from 90 participants [81], soft, rather than hard, lateral wedge insoles have also been found to be more effective.

An additional advantage of lateral wedge insoles is CoP control [45], although this feature has not been examined thoroughly in previous reports. As discussed, CoP excursion through the stance phase demonstrates a characteristic lateral curvature. This response is functional in leading the pronated ankle into a supinated position to efficiently oscillate mechanical energy from heel contact to toe-off. This deviation may, however, be associated with lateral balance disturbance and ankle inversion sprain [85]. Thus, eversion support has potential for injury prevention by regulating excessive lateral CoP excursion. Careful consideration is required when incorporating this feature into shoe-insoles to correct excessive Valgus knee so as not to disturb functional supination.

## 4. Concerns for Custom-Moulding

In populations with significant foot deformities, it may be difficult to wear conventional shoes and customised insoles may be required. Custom-made insoles can accommodate individual-specific foot shapes and maximise the contact area between the foot and insole [11,30]. This promotes foot pressure distribution, and for those with significantly deformed feet, customisation appears to be essential [86,87].

Caution is, however, required for custom-moulding for two reasons. First, every part of the foot does not contribute equally to weight bearing; for example, the mid-foot has been reported to have little responsibility [14]. It is, therefore, possible that some parts of the foot may be more vulnerable to foot pressure than others. The second caution is that customisation could reflect and accentuate negative foot control. If a misaligned foot is scanned and moulded without correction, progression of the deformity may advance due to inadequate foot posture and additional foot problems may arise as a consequence [29]. Lateral CoP excursion is associated with the risk of inversion sprain [34]. If a foot is susceptible to inversion sprain, for example, custom-moulding without CoP modification may not help or could even further increase the risk of injury. A lateral wedge assists foot pronation and reduces knee adduction moment, but the lateral wedge may not adequately assist the foot with excessive Valgus [45]. It can, therefore, be implied that a foot with excessive Valgus may benefit from a medial wedge rather than a lateral wedge. Furthermore, custom-moulding was found not to treat Hallux valgus by regulating hyperpronation of the subtalar joint during the later stance phase when weight is concentrated on the metatarsal area [35]. The results suggest, therefore, that custom-moulding alone may not correct inadequate ankle joint kinematics.

Although custom-moulding is important, the insole’s fundamental geometry should support optimum foot pressure control and energy efficient loading by providing adequate pronation–supination coupling. Ideally, the thin insole surface layer should be individual-specific, while the layers below can be rigidly constructed to assist optimum foot control. Telfer et al. [31] described the potential of 3D foot scanning and 3D printing for custom-moulding. This is a more finely-tuned approach to insole design, and future customisation of insoles and footwear is likely to take advantage of this technology. Other recent studies have also reported that 3D foot scanning could be an effective method for custom-made footwear despite further research being required to more comprehensively test the precision of this technique, including an increased number of tested subjects and wider measurement parameters to define the foot segment [88,89].

## 5. Gait Analysis for Insole Development

**Figure 5:**
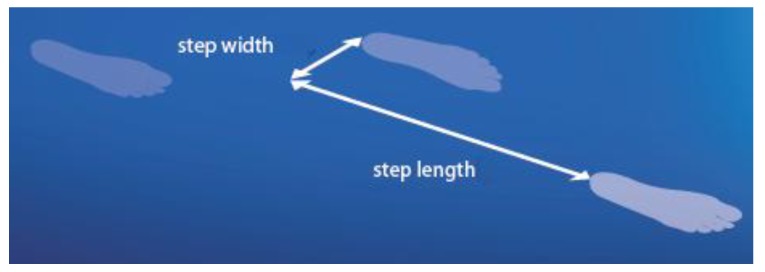
Spatio-temporal gait parameters, step length, and step width.

While shoe-insoles assist walking, detailed gait analysis has not been widely utilized to test their effects on gait patterns. While pressure data are instructive, as mentioned above, gait analysis can record human walking precisely to identify various problems, including the risk of falling and balance control and lower limb joint (ankle and knee) biomechanics problems. The utility of 3D gait analysis in identifying the causes of foot and knee pain has been discussed by Rao et al. [29], and Menant et al. [6] used these techniques to determine how footwear features influenced gait biomechanics. Heel collars, for example, also improve balance by providing increased tactile sensation around the ankle via the extended contact area provided by the collar, while a high collar was found to increase the risk of tripping by reducing swing foot height at MFC. In addition to identifying condition-specific biomechanical parameters, such as those associated with knee pain described above, spatio-temporal gait parameters also reflect walking fundamentals (Figure 5). For example, gait impairments due to injury, ageing, psychological conditions, or medications show similar gait patterns, including slower gait velocity (due to shorter step length), increased step width, and prolonged double support time [90]. Utilising 3D motion capture systems, the fundamental spatio-temporal gait parameters can be easily measured by markers attached only to the heel and toe. Alternatively, other gait assessment tools, such as the GaitRite mat, can also record stride cycle parameters. Motion capture systems have also been used for testing commercial products, such as footwear and anti-slip strips [91,92]. Commercially available 3D motion capture systems are now relatively affordable (e.g., Optitrack, NaturalPoint), and the complex programming for 3D gait analysis can be overcome using low-cost commercial software that automatically extracts a range of gait parameters given a pre-specified standardized marker setup at data collection.

## 6. Potential of a Wearable, Sensor-Integrated Insole for Real-Time Gait Monitoring

Digital gait analysis began using multiple standard video cameras to monitor human movement from various angles to estimate 3D motion. Later, 3D motion capture systems utilising either infrared light emitting diodes (active systems) or reflective markers (passive systems) provided highly accurate position-time data and are now widely used in experimental gait research. While these systems are used as the gold standard for gait assessment, limitations in this technology include a complicated setup, high time demands, a lack of portability, and a requirement for specialized skills for system operation and data analysis. In Section 5, potential directions to overcome these problems have been proposed, and one approach is wearable sensor technology, which, despite its limitations, receives considerable ongoing research attention due to its immensely practical application in gait measurement.

Small, inexpensive body-mounted inertial measurement units (IMU) can measure angular velocity and linear acceleration [93]. IMU data can also be transmitted to Android devices using Bluetooth [94]. These innovations create the potential for online management, such as mass data storage and automatic recording of personal gait data. Difficulties in utilising IMUs for gait monitoring remain in deriving position-time data due to complex noise filtering [93]. To date, a number of techniques have been proposed to obtain greater accuracy in estimating positional data but further efforts may be required until sufficiently reliable kinematic data estimation is achieved.

**Figure 6:**
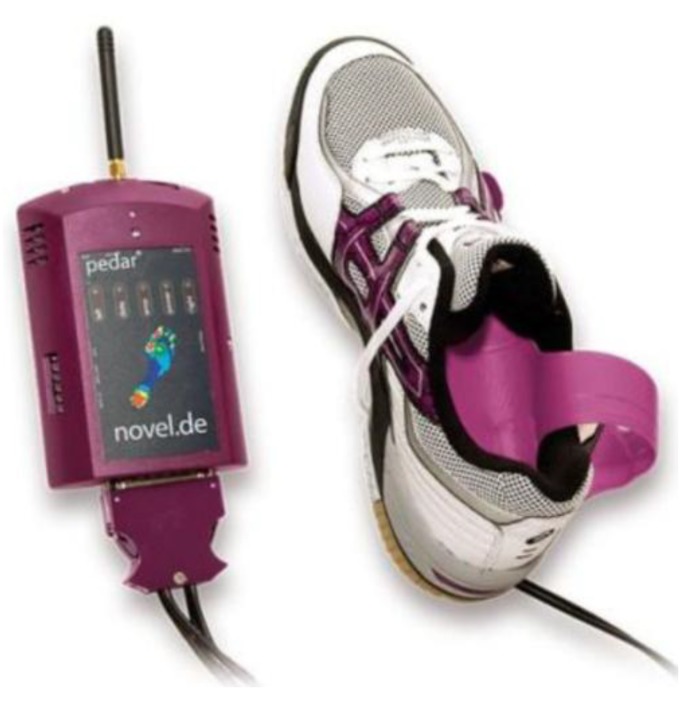
Foot-pressure monitoring system, Pedar (Novel, Munich, Germany, www.novel.de).

Incorporation of IMUs into an insole is a promising approach to gait management because IMU attachment to the foot has the advantage of concurrently recognising gait cycle events and, using them, estimating walking speed [95]. It is, however, still difficult to precisely estimate walking speed and other associated parameters, such as stride length, using IMUs alone. It is, accordingly, fruitful to consider adding other technology, such as foot pressure sensors (Figure 6) and global positioning systems (GPS) within the insole. In-shoe foot pressure sensors can more precisely detect foot contact than IMU data, while the GPS can track the walking path and, therefore, total distance travelled. In-shoe pressure monitoring insoles are available [10,96], and real-time monitoring can alert the user to inadequate pressure distribution, such as excessive plantar pressure, to prevent foot ulcers or identify a lateral CoP excursion warning of potential balance loss.

The VitaliSHOE project utilises wearable IMUs and pressure sensors integrated into the shoe-insole to detect the risk of falling in the senior population [97]. In an early paper, they showed the successful detection of transition between stance and swing based on both IMU and insole pressure data [97]. They identified, however, limitations due to gait measurement at a high walking speed and fragility of the IMU associated with mechanical stress [97]. In a later project [98], temporal variables were measured with high reliability but they also acknowledged the difficulties in acquiring reliable spatial data (e.g., step length) from IMUs [98]. It could also be interesting to utilise GPS technology to obtain spatial data in studies conducted in real-world settings, and smart shoes incorporating a mobile GPS have already been piloted as a fruitful direction for gait analysis in everyday settings [99].

## 7. Long-Term Effects

When developing shoe-insoles, the long-term effects of wearing them should be carefully evaluated to prevent overuse injuries, restriction of natural lower limb motion, and potential anatomical deformity [100]. Long-term use of a lateral wedge insole was found to reduce adductor moment semi-permanently, a positive adaptation; but if structured inadequately, adverse effects are possible [101]. As discussed earlier, foot control modifications can cause biomechanical changes to potentially all lower limb joint actions. Even very small negative features of the gait cycle can be accumulated by the thousands of steps taken every day and can eventually cause injury. Laboratory-based gait testing is useful in inspecting whether there are hazards in gait control. If gait kinematics and kinetics are maintained, it can be speculated that no negative long-term effects may arise. It is, however, still important to conduct human trials for a prolonged period to ensure that insoles are unlikely to cause orthopaedic problems.

## 8. Conclusions

Shoe-insoles have potential as an effective intervention to encourage safe walking. Biomechanical gait analysis is available for monitoring insole effects on walking performance. Insole modifications could support more adaptive ankle angles, improve foot pressure distribution, absorb shock, and reduce proprioceptive reaction time. Long-term effects of shoe-insoles should be tested to support and confirm experimental biomechanical evidence for their safety and effectiveness. Integration of wearable sensors into shoe-insoles will be a very important future direction for real-time gait measurement. Taking advantage of online data management, it will be possible to achieve the goal of detailed gait analysis available to everyone in performing the gait activities of everyday life.

## Figures and Tables

**Table 1:** Shoe-insole modification and biomechanical effects [11,12,13,14,15,16,17,18].

Modification Type	Potential Biomechanical Effects
Material	Shock absorption
	Pressure distribution
	Energy efficiency
Geometry (ankle control)	Shock absorption
	CoP
	Balance
	Energy efficiency
	Pressure distribution
Extra features (texture/heel cup etc.)	Reaction speed
	CoP
	Balance

CoP = centre of pressure.
